# Charge screening strategy for domain pattern control in nano-scale ferroelectric systems

**DOI:** 10.1038/s41598-017-05475-x

**Published:** 2017-07-12

**Authors:** Tomoaki Yamada, Daisuke Ito, Tomas Sluka, Osami Sakata, Hidenori Tanaka, Hiroshi Funakubo, Takahiro Namazu, Naoki Wakiya, Masahito Yoshino, Takanori Nagasaki, Nava Setter

**Affiliations:** 1 Department of Materials, Physics and Energy Engineering, Nagoya University, Nagoya, 464-8603 Japan; 2 PRESTO, Japan Science and Technology Agency, Kawaguchi, 332-0012 Japan; 3 Ceramics Laboratory, EPFL - Swiss Federal Institute of Technology, Lausanne, CH-1015 Switzerland; 4 Synchrotron X-ray Station at SPring-8 and Synchrotron X-ray Group, National Institute for Materials Science, Sayo, 679-5148 Japan; 5 School of Materials and Chemical Technology, Tokyo Institute of Technology, Yokohama, 226-8502 Japan; 6 Department of Mechanical Engineering, Aichi Institute of Technology, Toyota, 470-0392 Japan; 7 Research Institute of Electronics, Shizuoka University, Hamamatsu, 432-8561 Japan; 8 Department of Materials Science and Engineering, Tel-Aviv University, Ramat Aviv, 69978 Israel

## Abstract

Strain engineering is a widespread strategy used to enhance performance of devices based on semiconductor thin films. In ferroelectrics strain engineering is used to control the domain pattern: When an epitaxial film is biaxially compressed, e.g. due to lattice mismatch with the substrate, the film displays out-of-plane, often strongly enhanced polarization, while stretching the film on the substrate results in in-plane polarization. However, this strategy is of a limited applicability in nanorods because of the small rod/substrate contact area. Here we demonstrate another strategy, in which the polar axis direction is controlled by charge screening. When charge screening is maintained by bottom and top metallization, the nanorods display an almost pure *c*-domain configuration (polarization perpendicular to the substrate); when the sidewalls of the nanorods are metallized too, *a*-domain formation prevails (polarization parallel to the substrate). Simulations of the depolarization fields under various boundary conditions support the experimental observations. The employed approach can be expanded to other low-dimensional nano-scale ferroelectric systems.

## Introduction

The manipulation of domains in ferroic materials is a key in emerging functionalities of nano-scale devices^1^. In ferroelectrics, the orientation, size and distribution of the domains affect the dielectric, ferroelectric, and piezoelectric response^2^; therefore, the ability to control the domain structure is essential. However, available strategies for domain control in ferroelectrics are limited. In the case of thin films, a prominent approach is to control the crystal orientation and strain of the films by the selection of appropriate substrates^3–20^. The control of the cooling rate across a phase transition is used too for domain structure control^21^.

Electrostatic boundary conditions also affect the ferroelectric domain profoundly. They are the major reason for 180° domain formation: Without perfect charge screening, depolarizing field arising due to the polarization discontinuity at the surface of the film counteracts the non-screened polarization, resulting in antiparallel (180°) domain formation that reduces the energy of the depolarizing field^2, 22^. For example, epitaxial PbTiO_3_ thin films grown on SrTiO_3_ (STO) substrates show a fine 180° domain structure, such that overall, the bound charges self-compensate^23^. On the contrary, when a conductive substrate is used, a monodomain structure is maintained^24^.

In thin films, the interplay between electrostatics, elasticity, surface energy, and self-energy of domain walls can be utilized to obtain non-trivial patterns such as reconfigurable charged domain walls^25^, or linear arrays of <10 nm wide domains^26^. This is a topic of increasing research interest in thin films. In nanostructures, the subject is much less developed. Indeed, the polarization and domain patterns in nanostructures have been modeled intensively in recent years. An interesting work^27^ has demonstrated by phase-field modeling that adjusting surface charge screening can provide an efficient way to gain control of vortex domain structure in ferroelectric nano dots. However, experimental results rely mainly on the pioneering work of Schilling *et al*.^28^ who showed indeed morphological control of polar direction in ferroelectric nanowires, where changing the aspect ratio of nanowires, or changing their lateral dimensions locally resulted in local variation of the polarization direction.

The present study is focused on the role of charge screening in the control of domain pattern in nanorods and shows experimentally how to obtain *c-* or *a*-type domain orientation without the need of poling.

## Results and Discussion

**Figure 1:**
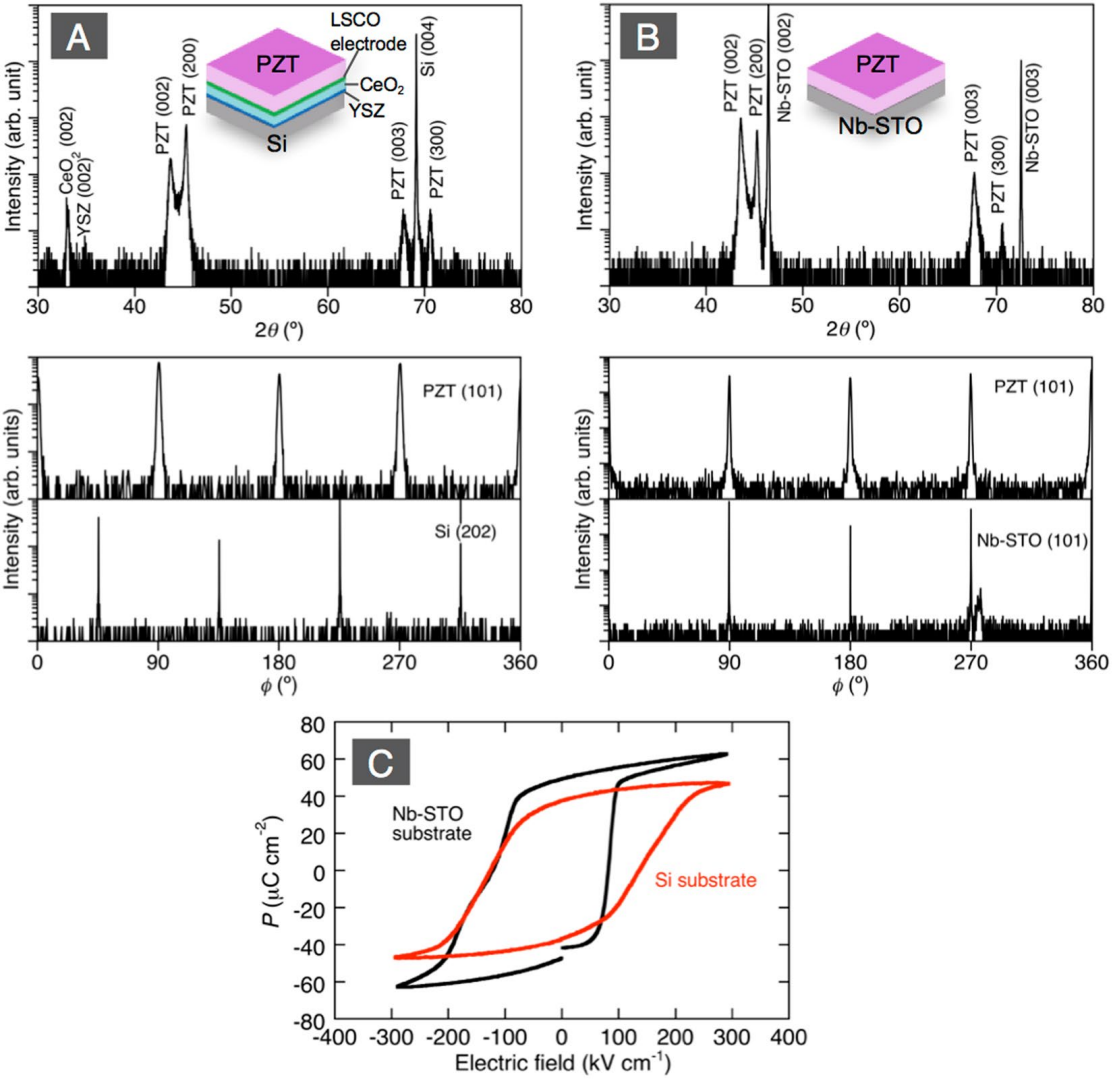
XRD patterns of PZT films deposited on LSCO/CeO_2_/YSZ-buffered on Si(100) (**A**) and Nb-STO (100) (**B**). The upper and lower plots show 2*θ*/*ω* and *ϕ* scans respectively. The *ϕ* scans are of PZT(101), Si(202), and Nb-STO (101). (**C**) shows the *P*-*E* hysteresis curves of the films.

**Figure 2:**
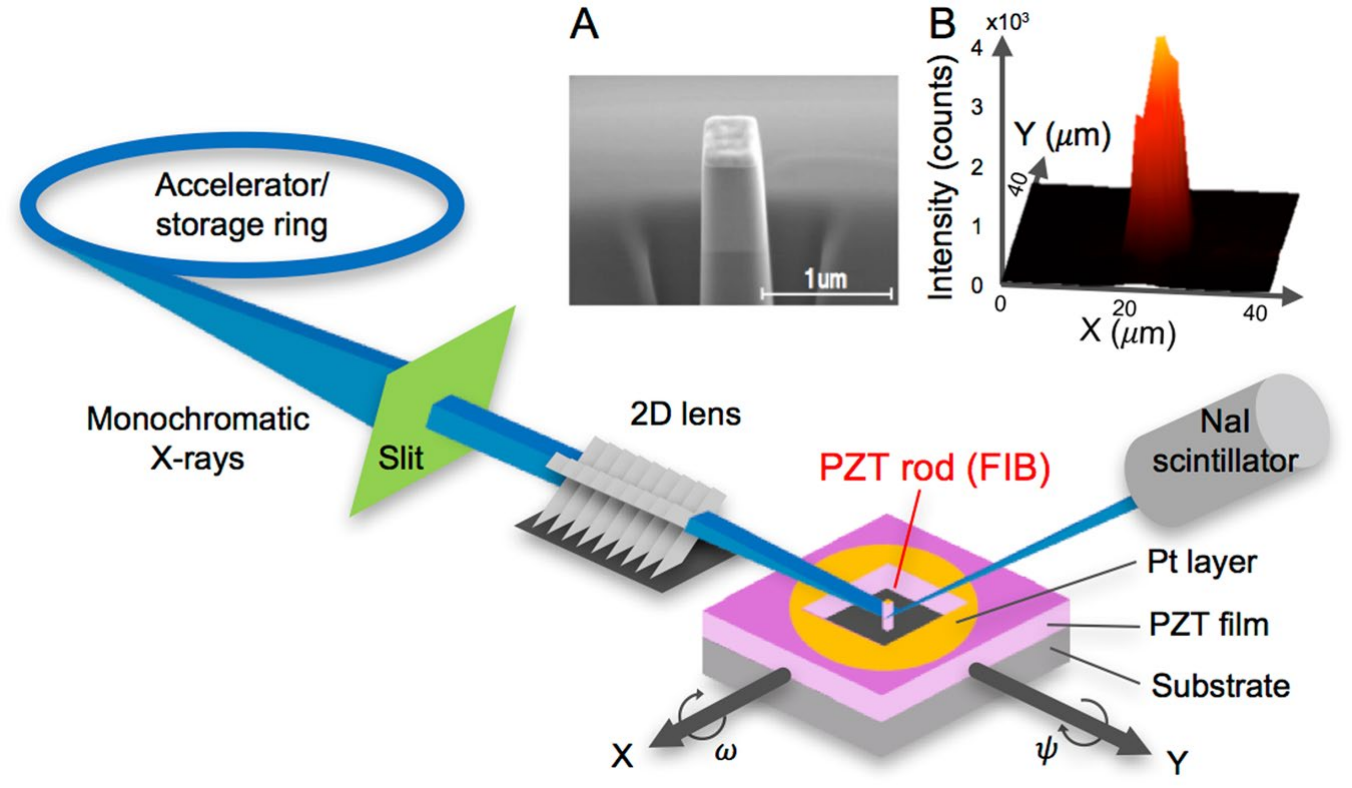
Schematic illustration of the set-up for synchrotron micro XRD build at BL13XU and BL15XU beamlines in SPring-8. X-ray beam was focused on a single rod fabricated by FIB. (**A**) shows a SEM image for 500 nm-wide rod, and (**B**) shows X-Y mapping for PZT (003) of 1 μm-wide rod, as typical examples.

**Figure 3:**
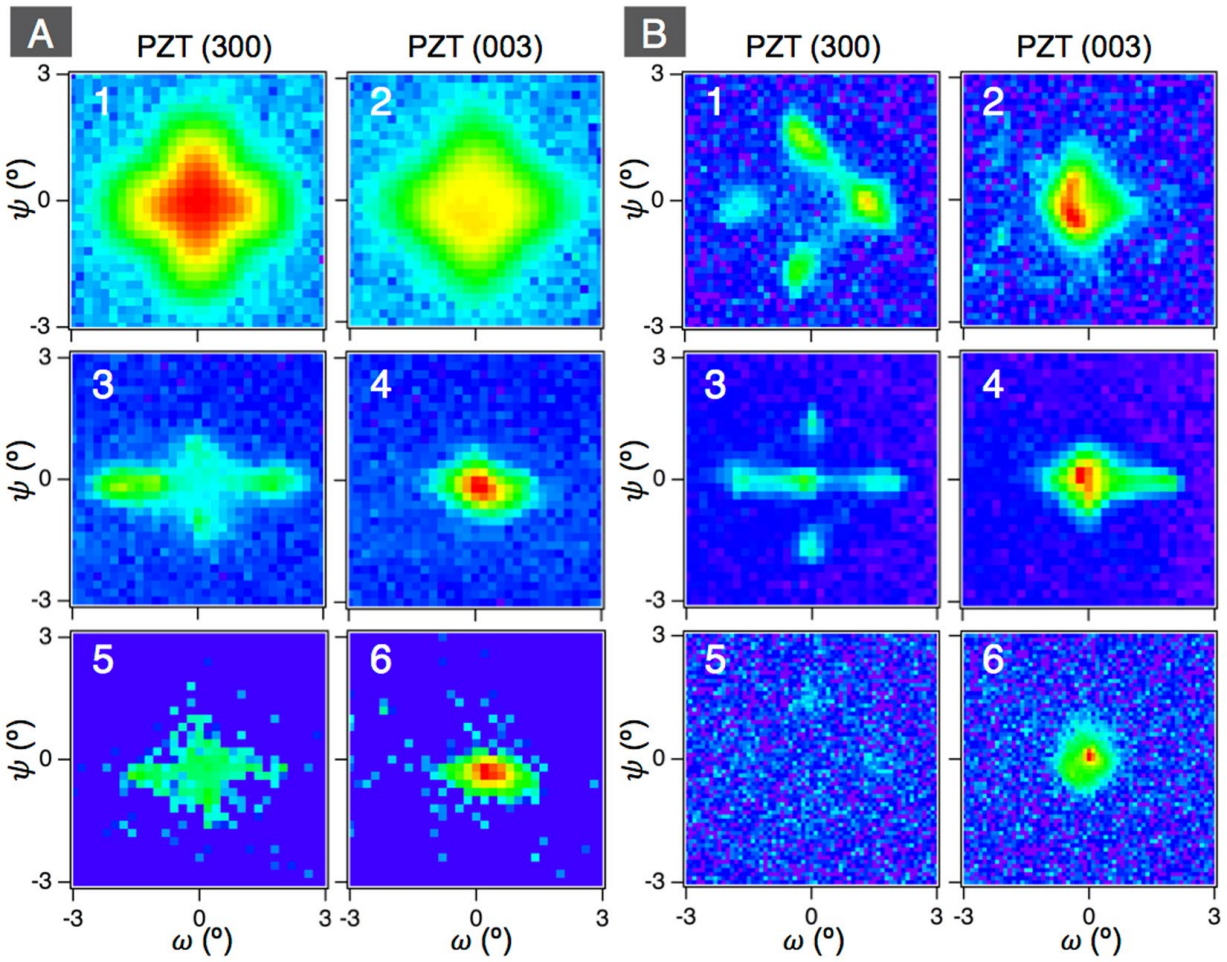
XRD *ω*-*ψ* maps for PZT (300) and (003) of the rods on LSCO/CeO_2_/YSZ-buffered on Si(100) (**A**) and Nb-STO (100) (**B**). 1 and 2, 3 and 4, and 5 and 6 show the films, the 2 μm-wide rods, and the 1 μm-wide rods, respectively. The maps for the rods on Nb-STO are quoted from ref. 29 (Copyright (2015) The Japan Society of Applied Physics).

**Figure 4:**
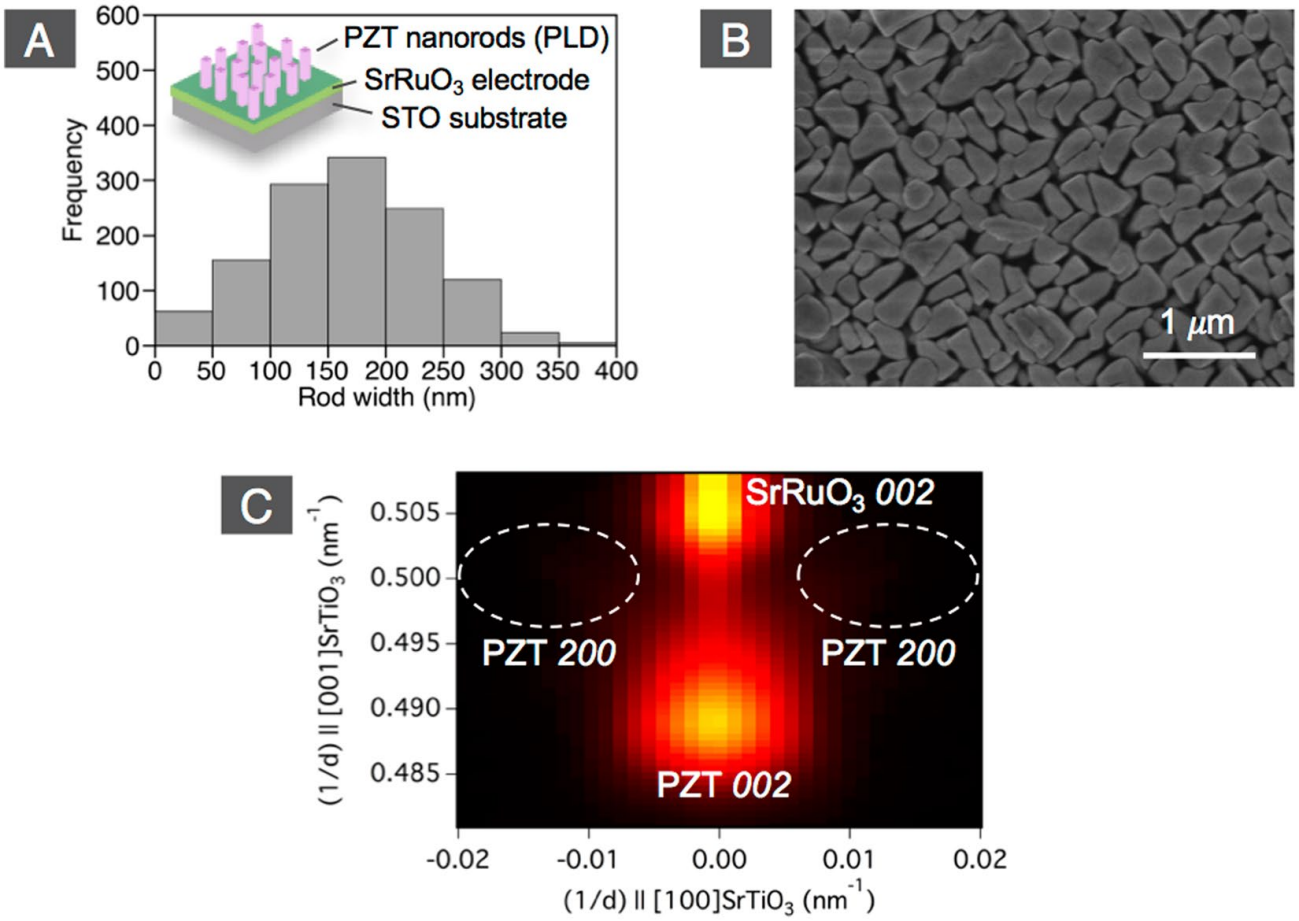
Self-assembled PZT nanorods grown on SrRuO_3_/STO(100) by PLD. (**A**) rod width distribution, (**B**) top-view SEM image, and (**C**) XRD reciprocal space map around PZT 002 and 200.

**Figure 5:**
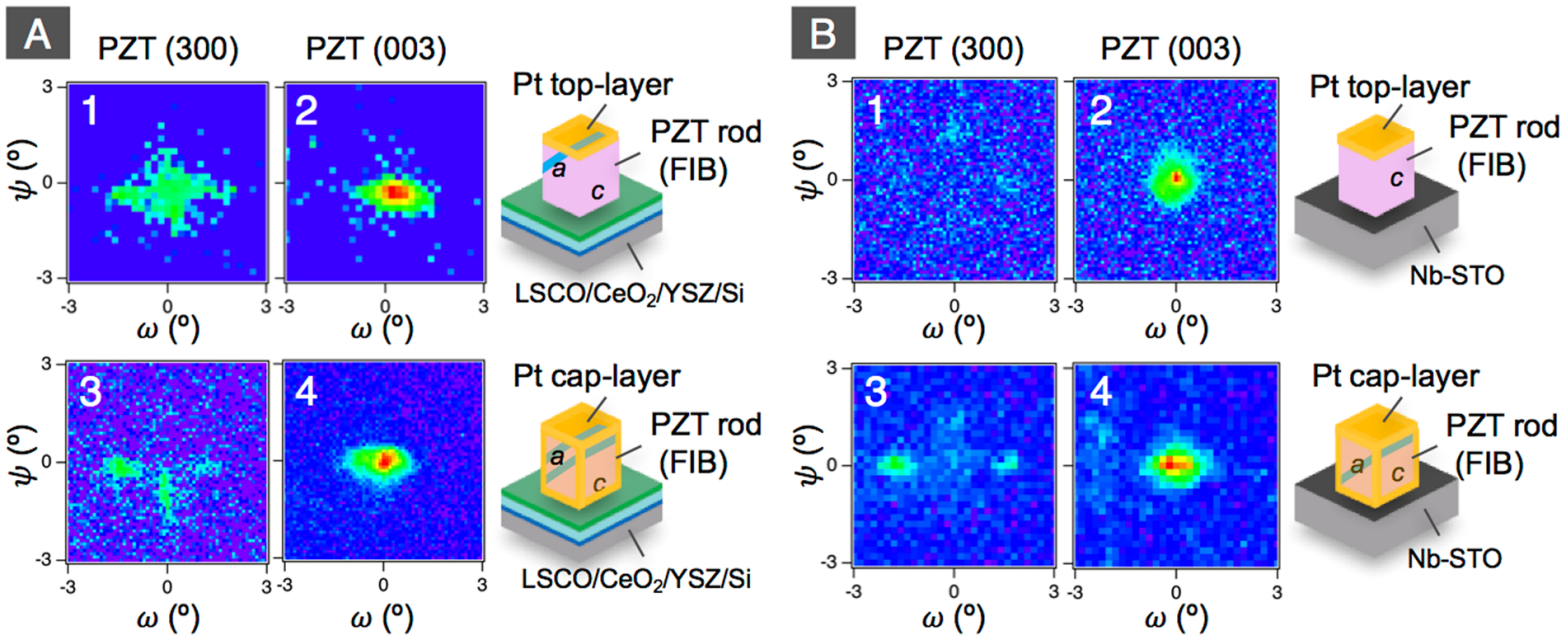
XRD *ω*-*ψ* maps for PZT (300) and (003) of 1 μm-wide PZT rods on LSCO/CeO_2_/YSZ-buffered on Si(100) (**A**) and Nb-STO (100) (**B**). 1 and 2: rod with top Pt-surface, and 3 and 4: rod covered with Pt on both top and sidewall surfaces.

**Figure 6:**
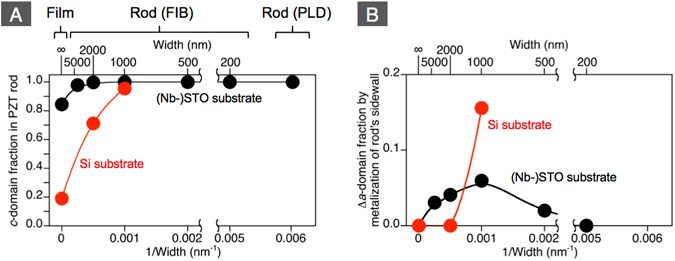
(**A**) *c*-domain fraction of PZT films and rods fabricated on LSCO/CeO_2_/YSZ-buffered on Si substrates and Nb-STO (or SrRuO_3_/STO) substrates as a function of the inverse rod width. The inverse rod width 0 represents the films. The fractions before the sidewall metallization by Pt are plotted. (**B**) increased amount of *a*-domain fraction by the sidewall metallization as a function of the inverse rod width.

**Figure 7:**
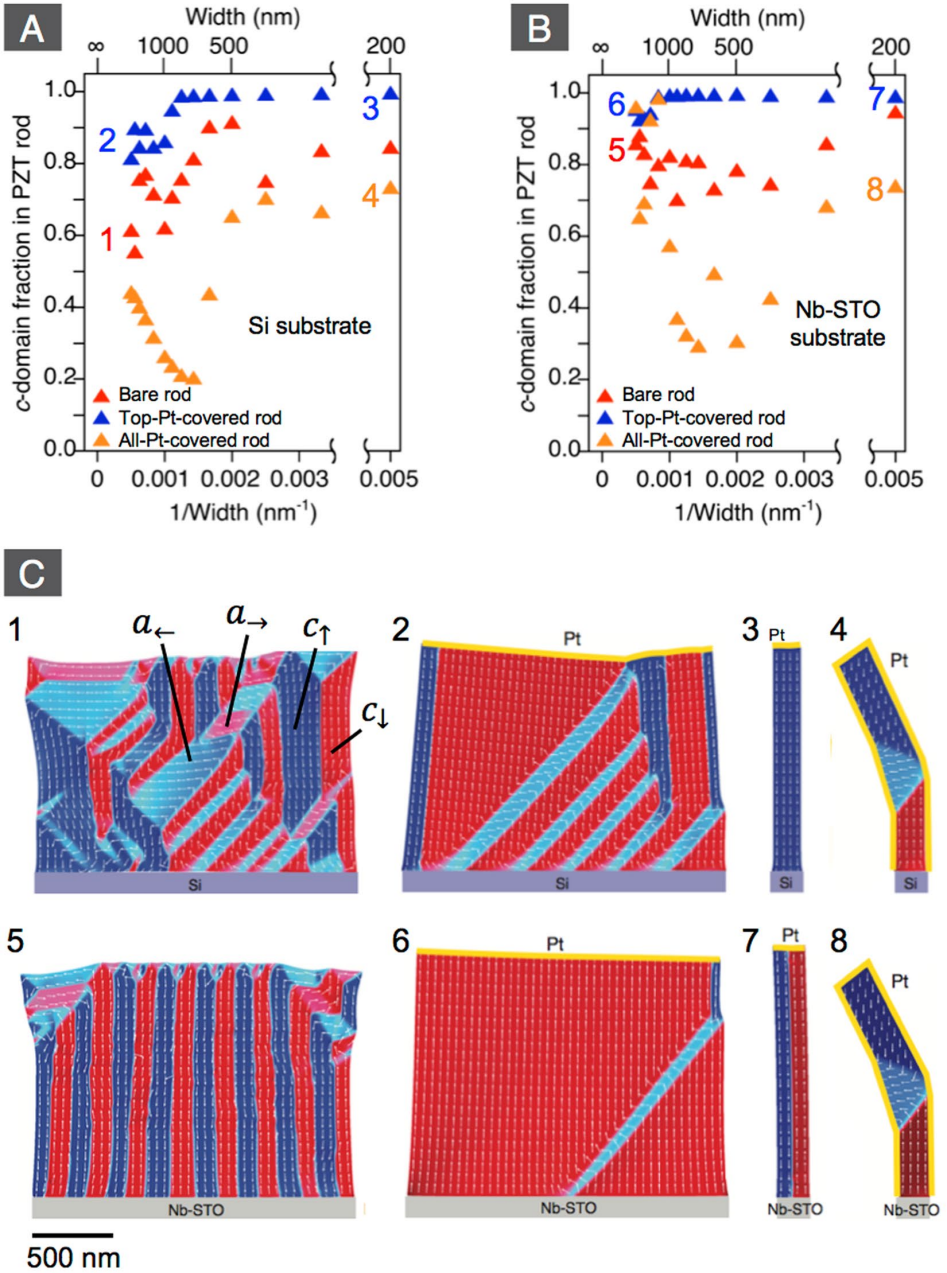
Calculated *c*-domain fraction of PZT rods on Si (**A**) and Nb-STO (**B**) by phase-field simulation. Red, blue, and orange triangles respectively show bare rods, rod with top Pt-surface, and rod covered with Pt on both top and sidewall surfaces. The numbers shown in (**A**) and (**B**) correspond to those in (**C**). (**C**) Calculated domain patterns of 2 µm wide rods with bare top surface on Si (1) and on Nb-STO (5), of 2 µm wide rods with top Pt-surface on Si (2) and Nb-STO (6), of 200 nm wide rods with top Pt-surface on Si (3) and Nb-STO (7), and of 200 nm wide rods with both top Pt surface and sidewall Pt surface on Si (4) and on Nb-STO (8).

The results are presented in the following manner: First we show the domain orientations in the as-grown {100}-epitaxial tetragonal-phase Pb(Zr_0.35_Ti_0.65_)O_3_ (PZT) films and the domain orientations in focused ion beam (FIB)-fabricated nanorods made of these films, which were grown on Si and Nb-doped SrTiO_3_ (Nb-STO) (Figs 1, 2 and 3). Due to the higher thermal expansion coefficient (TEC) of PZT relative to Si, the films grown on the Si are expected to be under tension, which is not the case when grown on Nb-STO. When grown on Nb-STO, the films, having a smaller TEC, are expected to grow in compression^14^. This will allow us to study the effect of charge screening and its interplay with the ‘interference’ imposed by the stresses in the film. Self-assembled nanorods grown on SrRuO_3_/STO substrate are used to confirm further those results (Fig. 4) and to discuss the interplay between the aspect-ratio of the nanorods and the depolarizing fields. Charge screening is further investigated by a series of experiments with nanorods having metallization on their sidewalls (Figs 5 and 6). Finally, the experimental results, namely the domain patterns in the nanorods as a function of their lateral size, substrate type and charge screening conditions are compared with phase field simulation of the various situations (Fig. 7) confirming the role of charge screening in the polarization orientation and domain pattern control.

Figure 1A and B show X-ray diffraction (XRD) patterns for 1200 nm-thick PZT films deposited on Si with epitaxial buffer layers of (La_0.5_Sr_0.5_)CoO_3_ (LSCO)/CeO_2_/yttria-stabilized zirconia with 8 mol % Y_2_O_3_ (YSZ) and on Nb-STO. The 2*θ*/*ω* scans showed that both PZT were grown with (100) and (001)-orientations, indicating the *a*/*c*-domain structure as normally observed in {100}-oriented PZT films. The *ϕ* scans for PZT (101), Si (202), and Nb-STO (101) indicated that the deposited PZT films have epitaxial relationships of PZT 〈100〉 || Si 〈110〉 and PZT 〈100〉 || Nb-STO 〈100〉, respectively. As can be seen in Fig. 1C, both PZT films showed well-saturated *P*-*E* hysteresis curves, though the measured remnant polarization values 37 μC/cm^2^ and 50 μC/cm^2^ for the films on LSCO/CeO_2_/YSZ/Si and Nb-STO were smaller than the theoretical spontaneous polarization 74 μC/cm^2^ of a single crystal due to the coexistence of *a*- and *c*-domains.

In order to clarify the domain structure of FIB-fabricated PZT nanorods, synchrotron micro XRD was employed. As illustrated in Fig. 2, we focused X-rays, whose beam size was about 4 μm × 4 μm at the full width half maxima, onto a single rod. The diffraction from a single rod was clearly separated from the film by the trenched areas (see Fig. 2B), which allowed us to investigate the domain structure of single rod quantitatively. Since {101} twin boundaries are formed in the *a*/*c*-domain structure of tetragonal ferroelectrics, the out-of-plane axes of *a*- and *c*-domains can be slightly tilted off the normal direction to the substrate surface. This allowed us to measure the diffraction intensities for the out-of-plane lattice spacing in *a*- and *c*-domains as functions of tilt angles, *ω* and *ψ*, with the rotation axes along Si [110] and [$1\bar{1}0$], and Nb-STO [100] and [010], respectively. Figure 3A shows the *ω*-*ψ* maps for PZT (300) and (003), representing the volumes of the *a*- and *c*-domains in the film and the rods with 2 μm- and 1 μm-widths on LSCO/CeO_2_/YSZ/Si. As can be seen in Fig. 3A-1 and 2, in the film the intensity for PZT (300) is larger than that for PZT (003). The estimated *c*-domain fraction is 0.19, which is less than the expected 1/3 for equilibrium-oriented domains without external fields and forces. Thus, the *a*-domain is dominant in the film grown on LSCO/CeO_2_/YSZ/Si. The reason for the preferred formation of *a*-domain is the mismatch of TEC between PZT and Si. Since TEC of PZT is larger than that of Si, PZT sustains increasing tensile stress during cooling down from the deposition temperature, which preferably generates the *a*-domain in order to minimize the elastic energy of the system. A similar domain structure of the films on Si has been also reported in the literature^13, 15^.

The domain fraction was drastically varied by forming the rod structure. As can be seen in Fig. 3A-3 to 6, for the rod structure, the intensities for PZT (300) are significantly smaller than that for PZT (003). The estimated *c*-domain fraction is 0.71 and 0.95 for 2 μm- and 1 μm-wide rods, respectively. It is thus observed clearly that while in the PZT film *a*-domains are predominant (>80%), the PZT rods fabricated from the film, have a predominantly *c*-domain structure.

PZT films grown on Nb-STO were under compression and hence were predominantly (80–90%) *c*-domain oriented, contrary to the >80% *a*-orientation in the films on the LSCO/CeO_2_/YSZ/Si substrate which were under tension. However, our previous study^29^ on the rods fabricated by FIB from PZT film grown on Nb-STO also showed similar features to the rods on the LSCO/CeO_2_/YSZ/Si substrate described above, with *c*-domains ratio increasing upon reduction of rod width, as shown in Fig. 3B. The comparison between the films and nanorods on LSCO/CeO_2_/YSZ/Si and those on Nb-STO precludes that the change in mechanical clamping plays an important role in the drastic change in domain orientation upon reduction of the rod width and is a key in the demonstration of the role of charge screening in domain orientation control.

We can also exclude a damage by the FIB process as the origin of the drastic change in domain fraction upon forming of the rod structures. Indeed, although we carefully etched the PZT with low acceleration voltages of ion beam, especially for finalizing the FIB process, a reduction of the piezo-response was observed due to the Ga injection and amorphousization at the vicinity of the surface. However, this was recovered by annealing the fabricated structure at the temperature ≥550 °C^30, 31^. Similar post-treatments have been also reported for recovering from the FIB damage in nanostructured ferroelectrics^32, 33^. This is further confirmed by our finding that self-assembled PZT nanorods grown on SrRuO_3_/STO(100) by pulsed laser deposition (PLD) without using any post-structuring showed as well the complete *c*-domain structure. As can be seen in Fig. 4A and B, although there is a wide distribution of the width in the self-assembled PZT nanorods, the average diameter of 166 nm is smaller than the rods fabricated by FIB. The XRD reciprocal space map (Fig. 4C) shows a clear PZT *002* peak from the *c*-domain but no PZT *200* peak from the *a*-domain. This confirms that a FIB damage is not the driving force for the drastic change in domain fraction that occurred by the rod structure formation.

In nanorods having aspect ratio much larger than one, the depolarizing field perpendicular to the nanorod is significantly larger than that parallel to it, hence formation of *a*-domain is prohibited. Indeed, the self-assembled PZT nanorods, whose average aspect ratio is much larger than one, had only the *c*-domain (Fig. 4). In the case of aspect ratio around one, the depolarizing fields perpendicular and parallel to the rods are similar, which stabilizes both *a*- and *c*-domains with the appropriate fraction according to the aspect ratio. The observed tendency of increasing *c*-domain fraction with decreasing rod width is explained by the depolarizing field. However, the *c*-domain was dominant even in the 2 μm-width rod on LSCO/CeO_2_/YSZ/Si (see Fig. 3A-3 and 4), with aspect ratio 0.6 < 1. This may be attributed to the different charge screening states at top and bottom interfaces and sidewalls of rods. In the rods fabricated by FIB in this study, the top and bottom interfaces are connected with conductive materials. In contrast, the sidewalls are exposed to the air. Although a certain degree of charge compensation would also occur on the sidewalls, that at the top and bottom interfaces is certainly larger. This asymmetric charge screening state have led to the preferable *c*-domain formation in this thicker rod.

To further investigate the role of charge screening as the major driving force in the control of the domain pattern, we deposited 10 nm-thick platinum layer on the sidewalls of the same rods that were measured by XRD. The rods were then heated above *T*_c_ and cooled back to room temperature. Figure 5 shows the *ω*-*ψ* maps for the 1 μm-width PZT rods on LSCO/CeO_2_/YSZ/Si and Nb-STO before and after the deposition of platinum layer on the sidewall. For both rods, the integrated relative intensity for PZT (300) increased by the metallization of sidewall. Although the change in domain fraction is not easily recognized by the naked eyes for the rod on LSCO/CeO_2_/YSZ/Si, the rod on Nb-STO clearly shows the appearance of *a*-domain in the sidewall metalized rod relative to the complete *c*-domain structure before the sidewall metallization. Figure 6A and B plot the *c*-domain fraction before the sidewall metallization, and the increased amount of *a*-domain fraction by the sidewall metallization as a function of the inverse of the rod width. As can be seen in Fig. 6B, the increase of *a*-domain is accelerated with decreasing rod width and is most appreciable when the rod width is 1 μm (namely, the aspect ratio is near 1). However, the narrower rods showed a lesser impact of the sidewall metallization. Indeed, the domain fraction was not obviously changed by sidewall metallization for the 200 nm-width rod on Nb-STO. The smaller impact of the depolarizing field on domain fraction in such a narrow rod is due to the appreciable depolarizing field perpendicular to the rod resulting from the finite screening length at the metal/ferroelectric interfaces perpendicular to the rod axis^34^.

In order to reinforce the experimental results, the observed results were compared with phase-field simulation for PZT rods on Si and Nb-STO substrates. In the simulation, we ignored the influence of lattice mismatch between PZT and substrate as the thickness of PZT was much larger than the critical thickness for misfit dislocations, but took into account the influence of thermal mismatch between PZT and substrate. We also assumed that the substrate surface is conductive as PZT was deposited on the conductive layers grown on Si and the conductive Nb-STO. As can be seen in Fig. 7A and B, the rods on Si basically show smaller *c*-domain fraction than those on Nb-STO due to the biaxial tensile stress. However, the *c*-domain fraction is always much larger than 1/3 when top and sidewall surfaces of the rods are not metalized (see the plots for bare rods in Fig. 7A and B). This is because of the better charge screening at the bottom interfaces; namely, the depolarizing field parallel to the rod, i.e. perpendicular to the substrate surface, is smaller than that perpendicular to the rod. When the top surface is metallized, the depolarizing field parallel to the rod weakens further. Therefore, the *c*-domain fraction is increased. The above, typical evolution of domain pattern can be seen when comparing Fig. 7C1 and C2, or Fig. 7C5 and C6. By reducing the rod width, the depolarizing field perpendicular to the rod increases. Thus, the *c*-domain fraction further increases as observed in the experiment (see also Fig. 7C3 and C7). In contrast, when the sidewall is also metallized, the *c*-domain fraction is reduced (and *a*-domain fraction increases) as the depolarizing field perpendicular to the rod weakens (Fig. 7C4 and C8). The impact of the sidewall metallization is largest when the aspect ratio is around 1. When the achieved charge screening at the sidewall is as high as that at the top and bottom interfaces, the *c*-domain fraction should decrease toward around 1/3 as shown in Fig. 7A and B. However, the experimentally observed *c*-domain fraction after the sidewall metallization was larger than the expectation for both rods on LSCO/CeO_2_/YSZ/Si and Nb-STO. A possible reason is the imperfect surface quality of sidewall. Although the annealing after FIB process drastically recovered the piezo-response, the surface of sidewall might have not been perfected on atomic scale, which would prevent the ideal reduction of the depolarizing field by the metallization. Nevertheless, the simulated patterns of domain evolution are qualitatively in excellent agreement with the experimental observation confirming that the domain pattern in ferroelectric rods can be controlled by charge screening.

## Conclusion

The influence of charge screening on the domain pattern for {100}-epitaxial PZT rods was investigated. The size-controlled tetragonal PZT rods were fabricated by etching PZT films grown on Si and Nb-STO substrates using FIB. It was found that the *c*-domain fraction increased with decreasing rod width, and the *c*-domain dominant structure was formed even in rods on Si substrate, which are under the tensile stress. In addition, by depositing Pt on the sidewall of rods, *a*-domain formation prevailed. All the observed results were explained by the depolarizing field, arising from the imperfect charge screening. The reduction of rod width enhances the depolarizing field perpendicular to the rods; on the contrary, the metallization of the sidewall of rods weakens it. Phase-field simulations fully supported the observed tendencies. That the domain pattern in ferroelectric rods can be controlled by the charge screening state was both experimentally and theoretically demonstrated. The proposed approach can be widely applied for tuning the performance of emerging piezoelectric nano electro mechanical systems such as nanoscale sensors and actuators.

## Methods

### Film Deposition

Tetragonal-phase (100)/(001) Pb(Zr_0.35_Ti_0.65_)O_3_ films of 1200 nm thickness were grown epitaxially on two type of substrates, (a) Si(100) substrates buffered with LSCO/CeO_2_/YSZ epitaxial triple layers, and (b) Nb-STO (100) substrates. All layers were deposited by PLD with KrF excimer laser (λ = 248 nm). The epitaxial triple layer template on Si consisted of 10 nm-thick YSZ, 20 nm-thick CeO_2_, and 20 nm-thick LSCO, whose deposition conditions can be found in ref. 35. For the growth of PZT, laser energy and repetition rate of 60 mJ and 7 Hz, respectively were used, at O_2_ pressure of 200 mTorr and temperature of 625 °C. To achieve the stoichiometric lead content in the deposited PZT films, a ceramic Pb (Zr_0.35_Ti_0.65_)O_3_ containing 5% excess PbO, sintered by spark-plasma sintering, was used as the target.

### Fabrication of Rods by FIB

Following the PZT film deposition explained above, circular platinum pads with 200 μm diameter and thickness 50 nm were deposited by electron beam evaporation, to serve as a protective layer for the FIB treatment which followed. The PZT film under the circular platinum pad was then etched into a rectangular columnar rod shape by the FIB, in a similar procedure to that reported in the literature^32, 33, 36, 37^. 80–100 μm squared area surrounding the rod was etched away to avoid XRD from the film. To shape its vertical walls, the roughly etched rod was irradiated by a FIB inclined from the normal to the PZT film by 5°. Rods of widths 200 nm, 500 nm, 1 μm, 2 μm, and 4 μm were fabricated (in this paper, the word “rod” is used for all the fabricated structures for simplicity, even when the aspect ratio is less than 1). The detailed structure was reported in refs 29, 30 and 31. For recovery from the etching damage and promotion of the formation of a stable domain structure, the PZT nanorods were annealed at 550–650 °C in air for 2 h.

### Self-assembled Growth of Rods by PLD

In order to verify that the domain structure formed in FIB-rods did not originate from a damage during FIB process, self-assembled epitaxial nanorods with similar thickness to FIB-rods were also prepared. These nanorods were grown on SrRuO_3_/STO(100) by PLD at elevated O_2_ pressure (2 Torr), which enhances the shadowing effect^38^ for PLD species due to their numerous scattering during the flight from target to substrate, resulting in the epitaxial growth of nanorods. The details of the growth and properties of the self-assembled nanorod will be described elsewhere.

### Structural and Electrical Characterizations

The crystal structure of the PZT films was determined by XRD using a four-axis diffractometer with Cu-*K*_α1_ X-rays (Bruker, D8 DISCOVER) and synchrotron X-rays (SPring-8, BL13XU beamline, Japan). Their ferroelectric properties were measured with a ferroelectric tester (Toyo Corporation, FCE-1). The microstructure of the fabricated PZT rods was observed by scanning electron microscopy (SEM). To investigate the domain structure of FIB-PZT rods, synchrotron micro XRD (SPring-8, BL13XU, and BL15XU beamlines, Japan) was employed. In these measurements, the X-ray beam was focused onto a single PZT rod centered on the square etched area using the 2D focusing lens system (Forschungszentrum Karlsruhe). We used X-rays with 12.4 keV photon energy (wavelength 0.1 nm) for these measurements. Due to the long duration of the measurements, the experiments were restricted to a single set of samples with different sizes. However, the contentious change of the domain fraction (see Fig. 6A) basically precludes an appreciable influence of local domains generated by particular morphology in each sample on the domain fraction. We also confirmed the reproducibility of domain fraction for the 1 μm-width rod on Nb-STO by measuring another sample. In addition, to reinforce the significance of the experimental results, the phase-filed simulations (described below) were performed.

### Phase-field Simulations

Phase-field simulations were performed and compared with the experimentally observed domain patterns. A two-dimensional phase-field model was adopted from ref. 39 and modified with Landau coefficients of (100) tetragonal Pb(Zr_0.4_Ti_0.6_)O_3_ from ref. 40, stiffness coefficients from ref. 41 and with the assumption of ideal dielectric properties with zero free charge. The bottom (100) boundary is assumed as ideally conducting in all cases and mechanically coupled to a purely elastic 1 μm thick substrate with assumed thermal lattice mismatch of 0.072% in case of Si and −0.336% in case of Nb-STO substrates. All other boundaries of PZT are mechanically free and with several combinations of surface metallization by Pt as described above. The substrate is clamped in vertical direction at the bottom and coupled via periodic boundary conditions in horizontal direction. The initial condition, which is introduced as a polarization noise and zero strain, is relaxed for PZT nanorods of horizontal dimension from 200 to 5000 nm in a time dependent solver of COMSOL 4.5a until stationary solution is reached.

### Data Availability

The authors declare that the data supporting the findings of this study are available within the paper.
